# Managing an automation development group

**DOI:** 10.1155/S1463924691000068

**Published:** 1991

**Authors:** S. D. Hamilton

**Affiliations:** Lilly Research Laboratories: a division of Eli Lilly & Co. Developmental Research & Technical Services Lilly Corporate Center Indianapolis Indiana 46285 USA


Journal of Automatic Chemistry, Vol. 13, No. 1 (January–February 1991), pp. 23–27

### Managing an automation development group*

**S. D. Hamilton**

*Lilly Research Laboratories: a division of Eli Lilly & Co., Developmental Research & Technical Services, Lilly Corporate Center, Indianapolis, Indiana 46285, USA*

### Introduction

In 1982, the Antibiotic Assay Department of Lilly Research Laboratories was in a situation similar to that experienced by other analytical laboratories: workload continued to rise steadily, while adding staff was becoming more difficult. Assays were shifting from more traditional wet chemical and biological analysis to chromatographic determinations. An increasing number of our new compounds required very extensive sample preparation prior to chromatographic analysis. A decision was made to add personnel to help cope with this situation by focusing on the automation of these operations.

This automation effort has now grown to a staff of eight people: the Automation Technologies group of the Fermentation Products Analytical Development Department. This group has developed nine different robotic sample preparation systems, seven on-line HPLC systems for real-time process measurement and control, six custom sample preparation units and five highly modified HPLC units to support a strain selection programme, replaced 10 segmented flow analysis systems with two highly automated centrifugal analysers and begun to master the art of real-time fermentor sampling, analysis and control.

The development and success of this group has resulted from a variety of factors. This paper examines these key factors and some issues that the automation effort has raised. An assessment of the impact of automation on the area is presented, along with a look into the future.

### Keys to success

*Strategic commitment—staffing*

The most important factor to the success of an automation effort is a management commitment to the strategic importance of automation. It must be strongly felt that automation is an essential component of the business unit's ability to perform its assigned role. Many people mistake willingness to spend money on hardware as a sign of management commitment. While this is undoubtedly important, the true test of an organization's desire to benefit from automation is the willingness to devote full-time staff to this pursuit. Custom automation efforts take time, study, attention to detail and endless communication with the 'customer'. Numerous areas of Lilly Research Laboratories have attempted to enter into custom automation projects without making this commitment, asking instead that scientists develop and sustain these efforts while performing their regular tasks. Almost without exception, these have eventually ended in disappointment. Many of these areas have now decided to 'wait for the market to bring the technology to them'. In other words, buy automation 'off the shelf' as it becomes available, rather than attempt custom automation projects. There is nothing wrong with this approach, provided that you can afford to wait and that a vendor does indeed market a device that meets your needs. However, the organizations that get ahead and make breakthroughs are usually not those that wait for solutions to be brought to them—they make their own.

*Knowledgeable and realistic expectations*

Too often, automation efforts are initiated as a search for the use of a given technology, rather than a search for automation needs. In the author's company, as laboratory robotics began to appear, many areas became sure that there was a role for robotics in their component, and they looked long and hard to find those opportunities. In this zeal to find a 'robotics application', not only were poor applications sometimes chosen, but viable, non-robotic opportunities were missed. A dedicated automation staff can be very knowledgeable about the variety of needs in its area, and, given time, will gather the necessary expertise to choose a solution that is appropriate to address the problem.

Automation groups should be customer driven, responding to the needs of the component they service. However, for a group just starting, it is wise to temper this drive with the need for the group to learn and develop. When beginning the automation effort in the Antibiotic Assay Department in 1983, the most pressing need was to automate the sample preparation done prior to HPLC analysis of Biosynthetic Human Insulin samples [1, 2]. This procedure was very lengthy, tedious, technique-dependent and used hazardous chemicals (CNBr): all excellent motivations to automate this process. However, it was not understood how to automate this very complex operation. Instead, the focus was placed on the assay of a similar product whose sample preparation required similar fundamental operations, but was much simpler overall [3]. The corporate need to automate this procedure was not as high, but was sufficient to provide justification. Fortunately, management saw the wisdom in this approach and allowed expertise to be built while addressing useful, if not highest priority, needs.

* Abstract published in *Journal of Automatic Chemistry*, Vol. 12, No. 6.

This same care must be taken when assessing the ability of the user area to absorb new technology. It cannot be realistically expected for a laboratory staff to cope overnight with a quantum leap of technology. Technicians who have never used an automatic pipette are probably not good candidates to operate a sophisticated automated system. A laboratory that lacks experience in instrumental techniques (i.e. HPLC, GC, FIA, IR, etc.) will not be able to support adequately something as complex as a robotic system. Such situations result in a very heavy support drain on the developing group, and usually generate discomfort with the user who feels loss of control of their process. Again, it may be necessary to set aside the highest priority needs and pursue those that provide a more gradual learning curve for the laboratory staff.

*Effective use of resources*

Developing laboratory automation is an interdisciplinary effort, requiring mechanical, electrical, programming and chemistry skills. While it may be possible to find individuals with some level of all these skills, at some point additional expertise will be needed. To develop an in-house laboratory automation group, it is necessary to hire people with non-traditional laboratory backgrounds. It could be, for instance, that an organic chemistry department might hire a mechanical or electrical engineer, or at least an organic chemist with a strong interest or second degree in mechanical engineering (ME) or electrical engineering (EE). The Automation Technologies Group has hired analytical chemists with strong instrumental backgrounds, and technicians with electrical engineering or computer science backgrounds.

No group can acquire all necessary expertise, so it is essential to identify key resources inside or outside the organization. The author's group, for instance, makes extensive use of a Lilly engineering group (MEs and EEs) whose primary role is to develop custom automation for production operations. This kind of engineering service is also available from a variety of commercial entities. Vendors of automation systems may offer custom fabricating and programming services. Other groups, often referred to as 'system houses' or VARs (value added remarketers), have capabilities to make special devices or assemble complete automated systems. In-house automation groups may at first be hesitant about using such resources, lest they be seen as less than expert themselves. In reality, if both parties have adequate experience, they can interact on a detailed level and benefit from the strengths and knowledge of the other. These collaborations have been found to work less well when there are large differences in the level of experience of each party.

A common mistake is to use these resource groups as a complete substitute for an internal automation effort. Such an approach does not lead to a knowledge-enhancing interaction for the buyer, so their organization becomes no smarter in the choice of, approach to or support of automation projects. The contracted automation system is often viewed as a 'black box' designed to perform a specific task. The user area must build a knowledge base about this technology, or the 'black box' is destined to be discarded when the original specifications no longer apply to the situation at hand. This would be similar to 'buying' a separation scheme when purchasing an HPLC, and discarding the instrument when that separation no longer was appropriate.

*Full project involvement*

One key to the success of an automation project is involvement in the full scope of project planning and execution. The above paragraphs address the need to carefully identify and evaluate user requirements, choose an appropriate approach and apply multidisciplinary expertise from a variety of sources. Some would consider then the delivery of a working device to be the concluding project phase for an automation group. The author's experience suggests otherwise.

The validation of the chemistry to be automated is perhaps the most time-consuming phase of projects. This is carried out as a joint effort with the eventual owners of the system, usually the group performing the manual version of the process. Invariably, unforeseen changes must be made both to the automated system and the chemistry, requiring the expertise and action of both groups. During this process the owners learn a great deal about the intricacies and operation of the system and the subtle interactions of the equipment with the chemistry being performed. Especially valuable to the automation developers is the direct experience about the performance of their creation and the modifications that must be made to perfect the operation.

Because of the author's group's proximity to its internal customers, it stays in close touch with an automation project as operation becomes 'routine'. Formal training classes are provided, when necessary, to make the owners more self-sufficient. Daily, long-term operation usually uncovers design oversights or component weaknesses. These must not only be corrected, but the knowledge of these occurrences must be assimilated for future reference. For instance, many of Lilly's robotic systems use bar-code scanning for sample identification. Initial testing, and the first two years of operation of such systems, indicated such a high level (>99.9%) of correct decoding that only basic provisions were built in to deal with the occasional non-decode. After several years, the printers began to age and suddenly there was not only an increase in non-decoded IDs but the occurrence of *incorrect* scans, i.e. generating the wrong number from the scan. This had originally been thought to be virtually impossible, and only the passage of time showed that it could happen. The systems now use an imbedded checksum to prevent incorrect scans.

Gathering this experience over the full scope of a project is only useful if people stay around long enough to apply their knowledge to further projects. Constant movement of staff is a way of life in many companies. Most of our automation projects span a year, and many involve two to three years. Three years is a minimum stay to experience enough project cycles to become proficient.

### Issues

As with other types of programmes, automation efforts generate issues that are not strictly under the control of the automation development group. The following are some of the issues that have arisen, and some of the ways they have been dealt with.

*Personnel*

The staff of the laboratory destined to take ownership of an automated system must not perceive this occurrence as a threat to their security. At Lilly there is absolutely no danger of anyone losing their job to automation, but people get very comfortable and secure in their routine and will naturally resist change. Efforts must start early to inform the staff about the goals of the work and get them as involved as possible in the planning, development and implementation. Clearly define how their role may change in the future and provide the necessary training to prepare for that change. It is usually not wise to generate great excitement about the arrival of the new automated system because the staff will resent so much attention being diverted to a 'machine'. Yet some extra attention is likely and (for those who have made an investment of time or money) usually necessary. It is best if the laboratory staff can be the conduit for this showcase, taking the role of the new 'expert' owners.

As more sophisticated automation is brought into an area, the general skill set of the staff must evolve. There will be less need for manipulative skills, and a heightened requirement for instrumental, computational and problem-solving skills. This may necessitate a change in hiring policy to attain the proper mix of skills. Five years ago, the Automation Technologies Group began hiring some laboratory technicians with two-year Electrical Engineering Technology degrees. These people are very comfortable using and troubleshooting highly automated systems. They are bright, quick learners, skilled at problem-solving and pick up the necessary chemistry knowledge easily. However, it has been found difficult to fight the lure of other, more electronics-oriented areas of the company, and these people unfortunatley tend to move on.

*Facilities*

As more and different types of automation were introduced, it was found increasingly difficult to place this equipment physically in the laboratory. Robotic systems required uniquely large, open square footage. Newer instruments and their PC-based controllers needed as much access to the back of the instrument as the front. Less bench space supplied with sinks, vacuum, steam and hoods was needed instead more wide, open floor space and bench tops on which to set instruments was wanted. These areas needed access to generous amounts of clean electrical power and communication connections. Typically, these changes were made as the need arose by removing, modifying or adding furniture, walls, pipes and wires. This approach was expensive and, more importantly, was repeated many times for the same space as needs continued to change. This led to the design of laboratories with a great deal of built-in flexibility, to accommodate and even encourage change. These facilities are of several designs, using such features as raised floors over flexible services, service drops from ceiling bulkheads, quick-connect services, movable walls and movable benches [4]. Since building these laboratories, many changes and modifications have been made to the use of the space. None have required any special construction or demolition, only labour to move flexible benches or walls and attach to or disconnect quick-connect services.

*Long-term support*

The inevitable result of a successful automation development group is the presence of a lot of custom instrumentation that must be supported. Just as we all expect commercial instrument vendors to provide on-going support for what they sell, internal customers also expect some level of support. Thus, as more automation is put into place, more total support time is required, reducing the time available for the primary development mission. Additional staff can be added, freeing more time for development, eventually leading to more support needs. The author has struggled with this cycle and has reached several conclusions:

What is delivered as a custom automated system is what the rest of the world might refer to as a 'beta test unit'. This is one step beyond a prototype, but still consists of many custom, or limited run, parts. The unit has gone through a good shakedown, but still has some rough edges and bugs left to be found. Beta units, by their nature, require much more support from the vendor than would a production model. Instrument manufacturers learn from their beta tests and move on to produce and sell many improved (usually), standardized replicates. This group at Lilly rarely gets to duplicate anything, so each creation remains a unique 'beta test' system, with its own special support needs. The laboratory is also a dynamic arena, and systems must often be changed to meet new needs. The conclusion is that in this environment, an on-going support role is necessary.

By vigorously educating users, the amount of time spent on support can be minimized. This takes time, not only to conduct classes but to prepare good teaching materials and plan useful exercises. Currently two levels of training are offered to Technical Service users. The 'system expert' course consists of over 40 h of in-depth hardware and programming training, and is designed to create a strong–pocket of expertise in that laboratory. The 'system operator' course lasts 4–6 h, and focuses on the rudiments of daily operation, maintenance and basic troubleshooting.

A mixture of maintenance support is necessary to deal with hardware failures. The first line of defence is in the users' lab. The training classes prepare the users to handle simple-to-moderate breakdowns. Users have dealt with problems such as leaky syringes or valves, dirty microplate reader optics, software bugs and communications lock-up. The next level of support requires someone who is able to do preventative maintenance checks and diagnose and repair moderate to major component failures. While the staff of the automation development group may be qualified to provide this service, it would detract from their primary role of development. This need is covered by a combination of in-house instrument maintenance shop and vendor service contract. As equipment becomes more complex, the instrument shop finds it increasingly difficult to remain proficient at component level diagnosis and repair. Yet service contracts do not guarantee repair by the next day. The instrument shop staff are well enough trained to diagnose most problems (in consultation with the user and/or vendor) and replace complete modules with spares on hand (purchased or obtained via service agreement). They may then attempt to repair these modules or return them. If they cannot solve the problems, then the vendor service expert is brought in. The training of shop staff is enhanced by having them work with the vendor service representative during repair or PM visits.

The most difficult part about the long-term support issue is encouraging users to depend on themselves, the instrument maintenance shop or the vendor. It is vital that users learn to be as self-sufficient as possible at solving their own problems or bringing the appropriate resources to bear to do so. In some cases the only way to motivate this development is to let users flounder a bit. This is usually unpopular, and it is wise to inform management beforehand about the strategy.

### Benefits

Ultimately, the worth of a dedicated automation effort is measured in the impact felt within the user area. These effects may take some time to develop, so one should not be too quick to pronounce judgement. As Lilly Research began its automation efforts, the common presumption was made that the main benefit of automation was going to be labour savings. This effect has definitely been felt, but there have been other, less tangible benefits of equal importance.

*Managing work-load change*

Automation systems have helped Technical Service's laboratories cope with increasing workloads. Figure 1 shows the increase in workload that the immunoassay

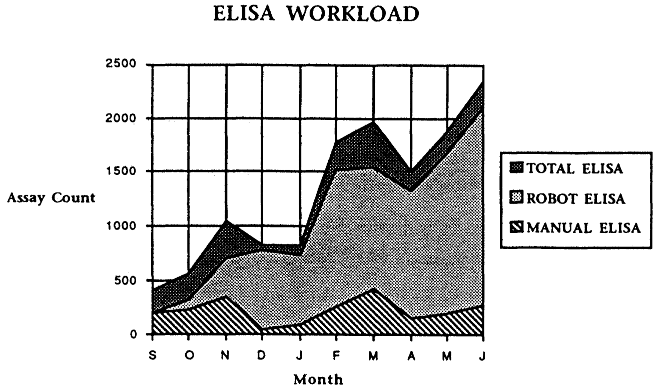

*Figure 1. Growth of departmental ELISA workload over a 10-month period. Virtually all of the additional workload was absorbed by the automated systems.*

group has absorbed using three robotic ELISA systems. They were able to ramp up smoothly their assay output without a major commitment of additional staff.

Workload change is not always characterized by growth, but by fluctuation. A constantly changing assay load can make management of an analytical laboratory difficult. Staffing must be adequate to cope with the peak demand periods, yet during slack times the staff must be gainfully employed. In a development facility, the workload may vary daily, with little advance notice. Automation has softened the impact of these fluctuations, by letting the automated systems absorb the peak workloads. During the slow periods, the equipment may be idle, but this is preferable to an idle person.

One example of this philosophy is the use of on-line HPLCs in the purification pilot plant. This facility develops large-scale chromatographic purification strategies, running several multistage experiments each week. In the past, hourly samples were taken from various points in the process and the final eluent was sampled and fractioned into drums. At the end of the experiment, all these samples were brought together to the analytical laboratory. Analysis results were needed quickly to plan the next purification experiment, so the laboratory would make a crash effort to meet this need. In between purification experiments, there was much less workload for this analytical lab. Anticipating these cycles is difficult because the pilot plant experiment schedule is not regular. By placing HPLCs on-line, directly measuring the purification process in real time, there is less need to send large numbers of samples to the analytical laboratory, so the laboratory operations are not radically affected by experimental scheduling.

*Flexibility*

Information about the concentrations of certain nutrients, inhibitors and/or metabolites is essential for fermentation process development. Technical Service's analytical laboratories have used, for many years, continuous flow analysers for these determinations. One instrument was dedicated to the analysis of a given species, i.e. glucose. Because this type of analyser requires at least 30 min to start up and equilibrate, an instrument would have to be left running all day to provide on-demand analysis, resulting in high maintenance requirements and considerable reagent consumption. At times, there were as many as 13 analysers in operation. Special, infrequent assay requests required 2 h to 1 day for setup, depending on equipment and manpower availability.

Several years ago, many of these determinations were adapted to be done on a random access centrifugal analyser. These automated systems have greatly increased our flexibility in responding to assay requests. These instruments are capable of storing 20 different reagents. The procedure for a given analyte may use one to three reagents. Any sample can undergo one, several or all of the determinations that the instrument is configured for, based on operator input. Sample load per run can range from one sample to 34. When no samples are present, the instrument waits in an idle mode, consuming only power.

*More timely data*

The process development area of Lilly Research Labs is a 24-h day, full-week operation. Samples that require analysis are generated throughout the day and night, but some lengthy analytical procedures are done only once per day on samples that have accumulated during the previous 24 h. Therefore, any given sample beginning analysis could be from 1 to 23 h old. Such was the case for the in-process analysis of Biosynthetic Human Insulin. A 12 h sample preparation was begun each morning at 8 a.m., completed at 10 p.m., with chromatographic analysis complete by 8 a.m. the next day. Assay turnaround (from time of process sampling) ranged from 1 to 2 days, depending on the age of the sample at the regular 8 a.m. start time of sample preparation.

By automating this process in a serialized way, the sample preparation/analysis process can be started at any time of the day that samples arrive. Therefore, assay turnaround time should not exceed 24 h, a benefit for making in-process decisions.

When very rapid assay turnaround is required, on-line analysis is used. Numerous real-time measurements systems provide immediate information to process control computers, which in turn make changes in the process operation. In these cases, the automated analytical laboratory is actually an instrument in the field.

*Rotation of staff*

Automation of intricate, lengthy or technique-dependent procedures can add to the ability of a laboratory to rotate and cross-train staff. Years ago, each fermentation development team did their own laboratory determination of cell mass in fermentation broth, a tedious process of multiple centrifuging, washing, pipetting and weighing steps. These data were important for evaluation of experimental runs. Each group had their cell mass expert, and there was great reluctance to change experts in the middle of lengthy studies. This made job rotation and cross-training difficult.

Automation of this procedure has removed the dependence on these 'experts'. The skills required are now much more mental (the ability to follow instructions), rather than physical technique. This allows the job to rotate freely among group members, based mostly on who needs the information. There are even cases where certain senior investigators find themselves without staff for the day, and manage to start and feed the robotic system themselves.

### The future

One way to effect the efficiency of process development is to provide a wealth of information rapidly when investigators find themselves at key decision points. This should help them evaluate process options better and faster, and lead to less dead ends due to lack of information. On-line analysis is a good way to provide this data, offering not only the means to closely monitor the process, but make real-time adjustments in key experimental parameters. Such measurements will continue to be a major focus of the author's group.

Progress is still being made in developing fully integrated automation solutions. Once information about a sample is manually entered into a computer somewhere, this process ought not need to be repeated with another system somewhere else. Thus the various computer systems, sample preparation and measurement automation need to talk to each other about where samples are and what needs to be done or has been done. Trying to build this around already existing computer and automation systems is a difficult task. At times the effort is not justified. Starting from scratch sounds easy when listening to vendors' sales talks, but it can be a daunting task.

In an area such as the author's analytical needs come and go quickly. Most of the laboratory automation efforts have focused on those needs that have remained around for some time. It is important to develop or find automation tools that can be brought to bear more quickly. This has happened in the computer world over the past 20 years, as the technology moved from large, inflexible mainframes to small, very flexible personal computers. Much of today's laboratory automation resembles a mainframe computer in concept: a large device that processes samples for a large number of people, through the care of a few well trained technicians. It is important to get to the stage of 'personal automation', where everyone has their own flexible and easy to use tools. Hopefully, the author's group will be able to play a role in this continued evolution.

### References

1. Gainer, F. E., in *Advances in Laboratory Automation Robotics 1985*, Eds J. R. Strimaitis and G. L. Hawk (Zymark Corporation, 1985), 100.
2. Hamilton, S. D., in *Advances in Laboratory Automation Robotics 1987*, Eds J. R. Strimaitis and G. L. Hawk (Zymark Corporation, 1987), 1950.
3. Hamilton, S. D., in *Advances in Laboratory Automation Robotics 1986*, Eds J. R. Strimaitis and G. L. Hawk (Zymark Corporation, 1986), 1.
4. Hamilton, S. D., *American Laboratory*, **19** (1987), 120.

